# Evaluating left atrial function changes by speckle tracking echocardiography in type 2 diabetes patients in Central Vietnam: a cross-sectional comparative study

**DOI:** 10.1186/s43044-024-00470-w

**Published:** 2024-03-28

**Authors:** Hai Nguyen Ngoc Dang, Thang Viet Luong, Toan Thanh Tran

**Affiliations:** 1 Duy Tan University, Da Nang, Vietnam; 2 Hue University of Medicine and Pharmacy, Hue, Vietnam; 3 Vietnam-Cuba Dong Hoi Hospital, Quang Binh, Vietnam

**Keywords:** Diabetes mellitus, Left atrial function, Speckle tracking echocardiography, Central Vietnam

## Abstract

**Background:**

Type 2 diabetes mellitus (T2DM) is a metabolic disorder that detrimentally affects multiple systems in the body, with a particular emphasis on the vascular and nervous systems. Despite its significant impact, limited studies have explored the influence of this condition on the left atrial (LA) function. To address this gap, our study utilized speckle tracking echocardiography (STE) to assess LA function in patients with T2DM in Central Vietnam.

**Results:**

The cross-sectional comparative study enrolled 134 subjects involving 66 patients with T2DM and 68 healthy individuals meeting the selection and exclusion criteria of the study. In our study, healthy individuals demonstrated higher values for LA reservoir strain (LASr), LA conduit strain (LAScd), and LA contractile strain (LASct), measuring 38.75% ± 5.43%, 19.58% ± 5.91%, and 19.16% ± 4.98%, respectively. In contrast, the T2DM group exhibited lower values for LASr, LAScd, and LASct, which measured 31.2% ± 4.56%, 14.77% ± 6.3%, and 16.36% ± 4.82%, respectively (*p* < 0.05). T2DM patients with normal LA volume index (LAVI) and normal left ventricular mass index (LVMI), LASr, LAScd, and LASct results were 32.07% ± 5.28%, 16.28% ± 6.95%, and 15.64% ± 5.32%. respectively.

**Conclusions:**

STE of the LA reveals a noteworthy reduction in reservoir, conduit, and contractile functions within the T2DM group when compared to the control group (*p* < 0.05). Furthermore, these impaired functions persist in T2DM patients even in the absence of increased LAVI and LVMI.

## Background

Diabetes mellitus is a metabolic disorder that causes damage to many systems in the body, especially the vascular and nervous systems. Globally, it stands as a significant health challenge, ranking as the third leading cause of death following cancer and cardiovascular disease. As of 2021, the International Diabetes Federation estimates approximately 537 million people worldwide live with diabetes, with projections indicating a surge to 643 million and 783 million by 2030 and 2045, respectively. In Vietnam, a 2015 STEPwise survey on non-communicable disease risk factors conducted by the Vietnam Ministry of Health revealed a national prevalence of diabetes and pre-diabetes at 4.1% and 3.6%, respectively, in the age group of 18–69. Updated data from the International Diabetes Federation in 2021 reports a 6.0% prevalence of diabetes among adults in Vietnam [1].

Diabetes predisposes individuals to various complications, with complex interplays of mechanisms such as hyperglycemia, insulin resistance, low-grade inflammation, and atherosclerosis. These factors collectively contribute to systemic effects, inducing structural and functional changes in the cardiovascular system [2]. According to the United Kingdom Prospective Diabetes Study, cardiovascular complications were present in up to 50% of patients at the time of type 2 diabetes mellitus (T2DM) diagnosis [3]. The complexity of treatment escalates when cardiovascular complications are detected concurrently with the disease. Therefore, the early identification of these complications in T2DM patients holds paramount significance.

While numerous studies have investigated early abnormalities in the structure and function of the heart in T2DM patients, the focus has primarily centered on the left ventricle (LV) and right ventricle [4–7]. Surprisingly, studies addressing the impact of diabetes on the left atrial (LA) function have been relatively scarce. Yet, the LA function serves as a robust predictive factor for various critical outcomes, encompassing atrial fibrillation, heart failure, stroke, and mortality [8, 9].

Several methods exist for assessing LA function, including cardiac magnetic resonance imaging, computed tomography, and cardiac scintigraphy [10]. However, these modalities pose challenges due to their expense, limited availability, and widespread implementation difficulties. In contrast, echocardiography emerges as an advantageous alternative, offering affordability, high safety, excellent temporal resolution, and the capability for repeated examinations. In recent times, STE, a technique facilitating myocardial deformation analysis, has emerged as a valuable tool for evaluating LA function. This assessment includes the LA's capacity to store blood (reservoir function), conduct blood, and pump blood (contractile function). Notably, changes in LA function have been established as an independent predictor of adverse cardiovascular events in individuals with T2DM [11, 12]. Despite the recognized significance of LA function, no study has investigated the role of STE in evaluating LA function, especially when the LV and LA structure appear normal in T2DM patients in Central Vietnam. Addressing this research gap, our study aims to explore LA function utilizing STE among T2DM patients in Central Vietnam.

## Methods

### Study population

The cross-sectional comparative study included 134 subjects who sought medical care at Hue University Hospital of Medicine and Pharmacy from April 2022 to July 2023. The study comprised 66 patients with T2DM in the patient group and 68 healthy individuals in the control group, all meeting the selection and exclusion criteria of the study. Informed consent was obtained from all study participants at the beginning of the study. The study protocol has been approved and endorsed by the Institutional Ethics Committee of Hue University of Medicine and Pharmacy (Code: H2022/309). The research was conducted following the guidelines stipulated in the Helsinki Declaration 2013.

#### Patients

This group consists of 66 patients diagnosed with T2DM according to the 2022 diagnostic criteria of the American Diabetes Association, including at least one of the following criteria: fasting plasma glucose (FPG) level of 126 mg/dL (7.0 mmol/L) or higher, or a 2-h plasma glucose level of 200 mg/dL (11.1 mmol/L) or higher during a 75-g oral glucose tolerance test (OGTT), or a random plasma glucose of 200 mg/dL (11.1 mmol/L) or higher in a patient with classic symptoms of hyperglycemia or hyperglycemic crisis, or a hemoglobin A1c (HbA1c) level of 6.5% (48 mmol/mol) or higher [13]. Furthermore, these patients had to meet the following criteria, including patients older than 35 years or adults with overweight or obesity (Body mass index (BMI) ≥ 23 kg/m^2^) who had one or more of the following risk factors: a family history of T2DM in a first or second degree relative, high-density lipoprotein cholesterol (HDL-C) level < 35 mg/dL (0.90 mmol/L) and/or a triglyceride level > 250 mg/dL (2.82 mmol/L), women with polycystic ovary syndrome, physical inactivity, and other clinical conditions associated with insulin resistance (e.g., severe obesity, acanthosis nigricans). Exclusion criteria encompassed patients with hypertension (blood pressure ≥ 140/90 mmHg), cardiac pathologies (severe aortic stenosis, moderate to severe aortic regurgitation, severe mitral valve stenosis, congenital heart disease, myocardial ischemia, reduced ejection fraction, arrhythmias, and chronic atrial fibrillation), implanted pacemakers, pregnant or lactating women, patients with acute life-threatening medical conditions, and cases with unclear or inadequate ultrasound images hindering accurate visualization of the cardiac myocardium.

#### Normal control

This group consists of 68 healthy subjects undergoing routine health check-ups, with no history of diabetes or cardiac pathology, and currently free from both cardiac and diabetic conditions. The control group is age-matched with the patient group. Exclusion criteria encompass individuals with unclear or inadequate ultrasound images that hinder accurate visualization of the cardiac myocardium.

### Data collection protocol

Data collection was conducted using ultrasound machines, paper medical records, and data collection forms to extract information from the study subjects. This comprehensive process encompassed the following steps:

#### Clinical examination

All participants were thoroughly informed about the benefits and risks of participating in the research and provided their consent for data extraction. The patient’s medical history was subsequently gathered, emphasizing factors such as the time of T2DM diagnosis. Height and weight measurements were conducted meticulously, with weight measurements accurate to 0.5 kg and height measurements precise to 1 cm. BMI was calculated using the formula: BMI = weight(kg)/[(height(m)]^2^ [14]. Body surface area (BSA) was calculated using the Du Bois formula: BSA = 0.007184 x (weight)^0.425^ × (height)^0.725^ [15]. Blood pressure measurements followed the recommendations of the American Heart Association in 2019 [16].

Blood tests: Blood samples for biochemical analysis were collected from fasting venous blood upon waking. The biochemical tests were executed using the Roche Cobas E601 automated clinical chemistry analyzer at the Central Laboratories Unit of Hue University of Medicine and Pharmacy Hospital.

#### Transthoracic echocardiography procedure

Our study employed the specialized Affiniti 70 ultrasound machine from Philips, the Netherlands, which simultaneously recorded electrocardiographic signals during imaging. Measurements adhered to the recommendations outlined in the transthoracic echocardiographic examination guidelines for adults as per the American Society of Echocardiography [17].

#### Speckle tracking echocardiography

**Fig. 1:**
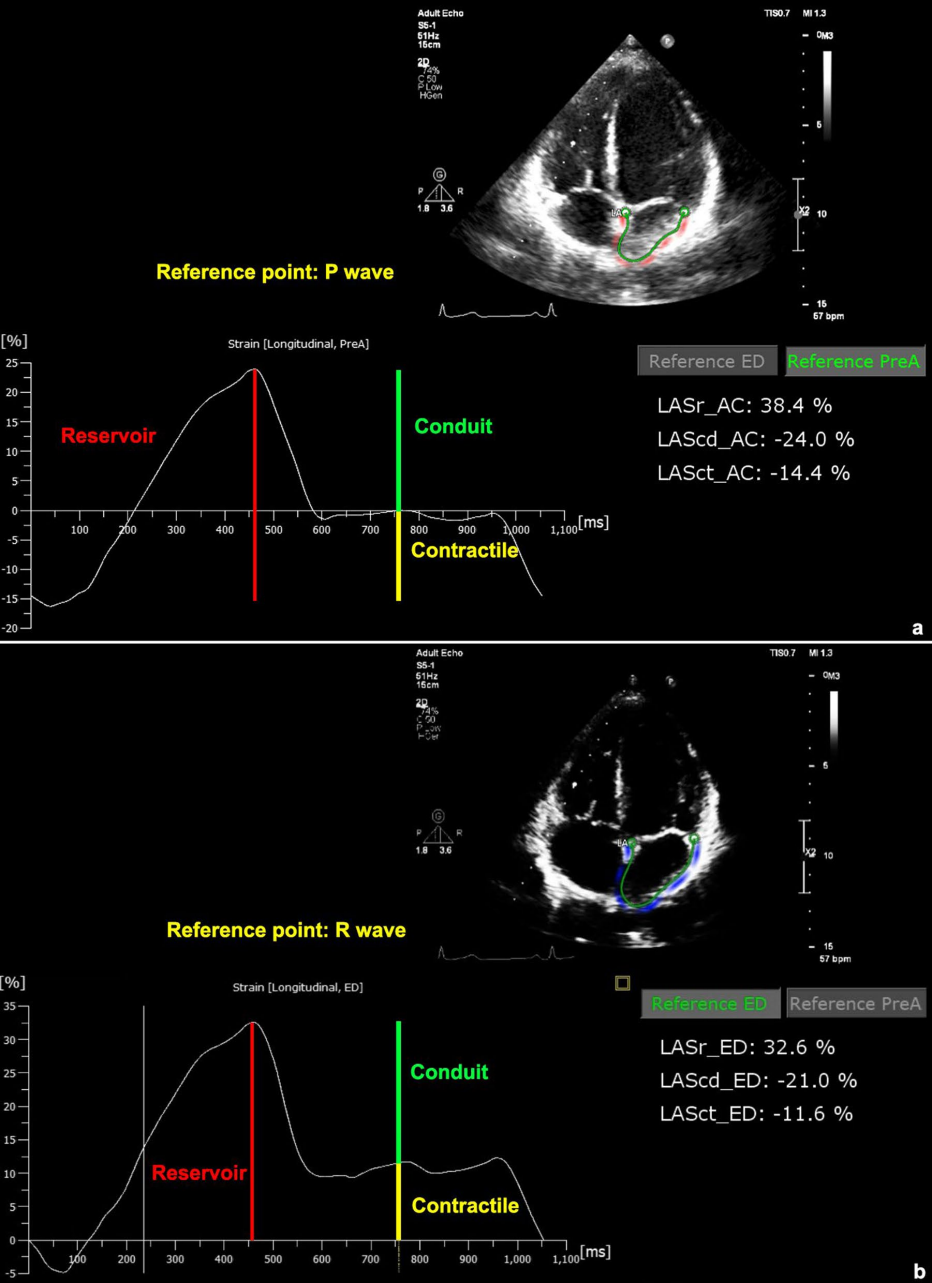
LA function values on STE. **a**: LA strain in the reservoir, conduit, and contractile phases were calculated by using the starting points of P-wave onset (LASr_AC, LAScd_AC, and LASct_AC, respectively); **b**: The LA strain values were calculated by using the starting points of R-wave peak (LASr_ED, LAScd_ED and LASct_ED, respectively)

Image acquisition involves the patient lying down with arms raised above the head to widen the intercostal spaces. The patient is tilted 90 degrees to the left relative to the bed, and 2D images are acquired at a frame rate of 60–90 frames per second, capturing a longitudinal section beside the chest. Subsequently, the patient is tilted about 30–40 degrees to the left, the transducer is positioned at the apex of the heart, directed towards the base of the heart, and images of the four and two chambers are acquired, each with one image per cut. The longitudinal section must pass through the apex of the heart. Each image is taken over two consecutive heart cycles, and then the images are saved to a Universal Serial Bus drive. For strain analysis, the cardiac muscle is analyzed using offline QLAB version 15 software. The endocardial border of the LA is identified through the point-and-click technique on the LA endocardium, performed by simply pointing and clicking on the screen. The software then automatically traces the surface of the LA endocardium, generating a region of interest adjusted to encompass the entire thickness of the heart muscle throughout the cardiac cycle. The automatic output of values for the LA function is provided by the software, based on reference points. These values include LASr_ED (%), LASr_AC (%), LAScd_ED (%), LAScd_AC (%), LASct_ED (%), and LASct_AC (%). The obtained results are absolute averaged to derive values for LASr, LAScd, and LASct. All strain measurements were performed by one strain specialist in the core laboratory who was blinded to the patients’ other data. The detailed methodology is illustrated in Fig. 1.

#### Statistical analysis

All statistical analyses were performed using IBM SPSS Statistics version 26.0. The normal distribution of variables was assessed by Kolmogorov–Smirnov test. Accordingly, continuous variables are expressed as mean ± standard deviation, if normally distributed, and as median (I and III quartiles) otherwise; categorical variables are reported as percentages. Organized research results into tables and charts. Utilized the Independent t-test to compare two mean values when two conditions were met: (1) Quantitative groups follow a normal distribution, (2) Homogeneity of variances across groups. Statistical significance was considered when *p* < 0.05 and highly significant when *p* < 0.001. The Mann–Whitney U test was employed to compare two mean values. This choice was made when the conditions for employing the Independent *t* test were not met, specifically when quantitative groups did not adhere to a normal distribution or lacked homogeneity of variances across groups. Spearman’s correlation coefficient was applied for non-normally distributed variables, while Pearson’s correlation coefficient was used for normally distributed variables to assess the correlation between continuous variables.

## Results

The study comprised 134 subjects who met the research criteria, and the baseline characteristics of the study group are detailed in Table 1. The mean age did not significantly differ between the two groups (*p* > 0.05). Furthermore, BMI, BSA, and waist circumference displayed no notable distinctions between the patient and control groups (*p* > 0.05). The average HbA1c concentration in the patient group was 11.26% ± 3.07%, with an average duration of diabetes detection recorded at 6.68 ± 5.16 years.

**Table 1:** Biometric characteristics of the study subjects

Variable	T2DM group (n = 66)	Control group (n = 68)	*p* value
Age (years)	60.11 ± 9.10	60.03 ± 5.89	0.954
Female	44 (66.7)	36 (52.9)	0.105
BSA (m^2^)	1.54 ± 0.14	1.55 ± 0.12	0.826
BMI (kg/m^2^)	22.05 ± 3.06	21.88 ± 2.03	0.703
WC (cm)	75.61 ± 9.42	76.32 ± 4.82	0.582
SBP (mmHg)	123.30 ± 10.04	120.81 ± 7.31	0.102
DBP (mmHg)	73.20 ± 8.29	74.71 ± 7.53	0.272
Duration of diabetes (years)	6.68 ± 5.16		
HbA1c (%)	11.26 ± 3.07		

Values are presented as mean ± standard deviation or number (%). Abbreviations: *T2DM*, Type 2 diabetes mellitus; *BSA*, Body surface area; *BMI*, Body mass index; *WC*, Waist circumference; *SBP*, Systolic blood pressure; *DBP*, Diastolic blood pressure; *HbA1c,* Hemoglobin A1c

Baseline echocardiographic assessments of the study group are delineated in Table 2. In conventional echocardiography, patients with T2DM showed higher values than the control group for LVM (161.92 ± 45.13 vs 148.90 ± 41.97), LVMI (104.64 ± 27.12 vs 95.64 ± 23.59), and IVSd (1.03 ± 0.15 vs 0.93 ± 0.14). The left ventricular ejection fraction (LVEF) in the patient and control groups was 68.30% ± 7.18% and 70.49% ± 5.93%, respectively. The values of LA functions assessed by STE in T2DM patients were significantly reduced compared to the control group, demonstrating statistical significance (*p* < 0.05).

**Table 2:** Echocardiographic parameters

Parameter	T2DM group (n = 66)	Control group (n = 68)	*p* value
LVM (g)	161.92 ± 45.13	148.90 ± 41.97	0.086
LVMI (g/m^2^)	104.64 ± 27.12	95.64 ± 23.59	0.042
LVIDd (cm)	4.51 ± 0.53	4.62 ± 0.48	0.205
IVSd (cm)	1.03 ± 0.15	0.93 ± 0.14	< 0.001
LVPWd (cm)	0.99 ± 0.15	0.91 ± 0.13	0.002
LAd (cm)	3.14 ± 0.41	3.08 ± 0.47	0.402
LAVI	19.38 ± 4.4	17.75 ± 6.51	0.099
LVEF (%)	68.30 ± 7.18	70.49 ± 5.93	0.056
LASr (%)	31.20 ± 4.56	38.75 ± 5.43	< 0.001
LAScd (%)	14.77 ± 6.3	19.58 ± 5.91	< 0.001
LASct (%)	16.36 ± 4.82	19.16 ± 4.98	0.001

Values are presented as mean ± standard deviation. Abbreviations: *T2DM*, Type 2 diabetes mellitus; *LVM*, Left ventricular mass; *LVMI,* Left ventricular mass index; *LVIDd,* Left ventricular internal diameter in diastole; *IVSd*, Interventricular septum thickness in diastole; *LVPWd*, Left ventricular posterior wall thickness in diastole; *LAd,* Left atrial diameter; *LAVI,* Left atrial volume index; *LVEF*, Left ventricular ejection fraction; *LASr*, Left atrial reservoir function; *LAScd,* Left atrial conduit function; *LASct,* Left atrial contractile function

**Fig. 2:**
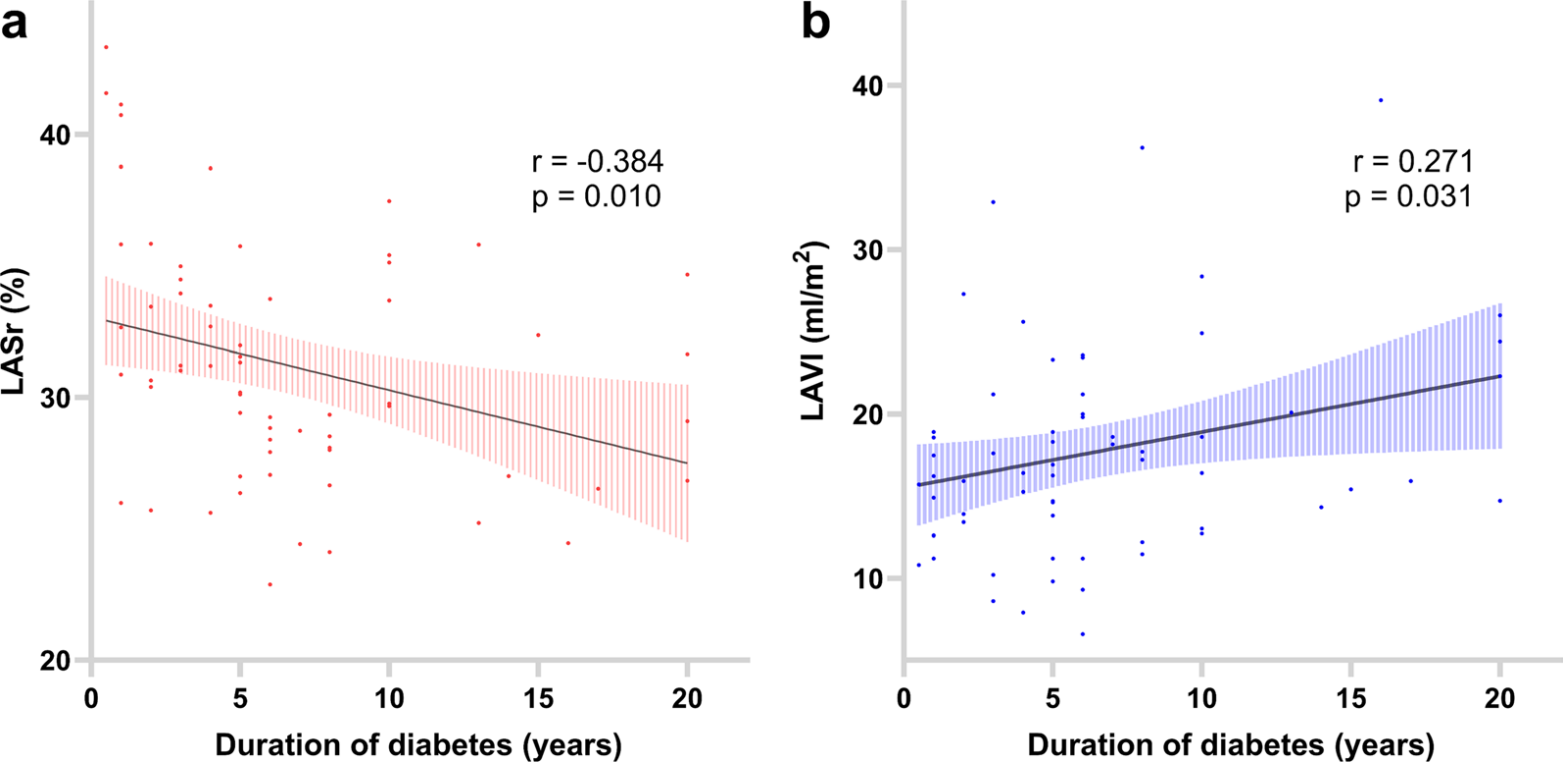
Correlation plot depicts diabetes duration's impact on LA function and morphology. Panel **a**: LASr demonstrates an inverse correlation with the duration of diabetes. Panel **b**: LAVI shows a direct correlation with the duration of diabetes

Our research findings indicate an inverse correlation between LASr and the duration of diabetes, with r = − 0.384 and *p* = 0.001. Conversely, LAVI shows a direct correlation with the duration of diabetes, with r = 0.271 and *p* = 0.031. Further details are illustrated in Fig. 2.

**Fig. 3:**
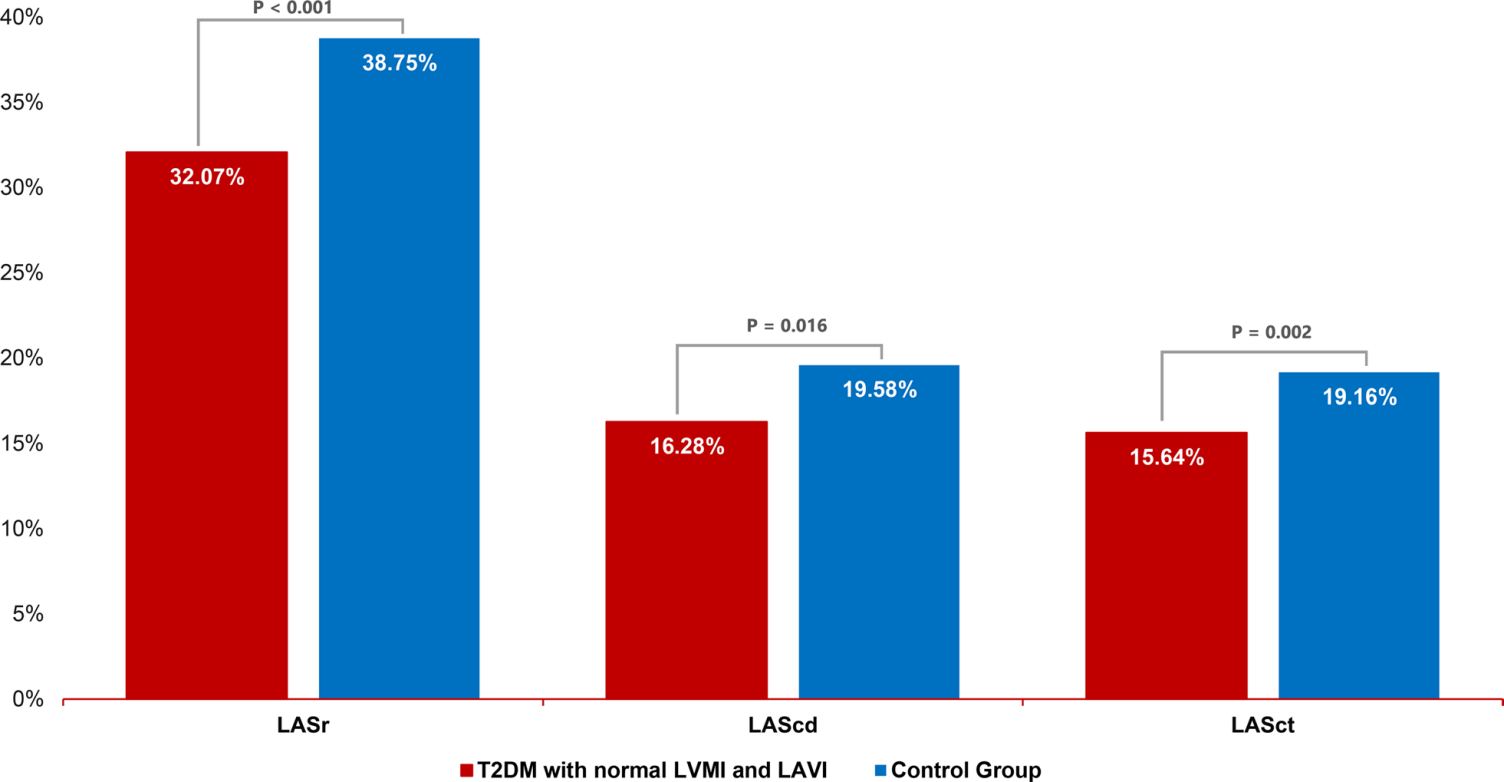
Mean values of LA strain on STE between the control group and the T2DM group with normal LVMI and LAVI

Table 3 and Fig. 3 illustrate the LA strain indices in patients with T2DM. Remarkably, even in the absence of abnormalities in LV and LA morphology, these indices exhibited a statistically significant reduction compared to the control group.

**Table 3:** LA strain in T2DM patients with normal LVMI and LAVI compared to the control group

Parameter	T2DM with normal LVMI and LAVI (n = 32)	Control Group (n = 68)	*p* value
LASr (%)	32,07 ± 5,28	38,75 ± 5,43	< 0.001
LAScd (%)	16,28 ± 6,95	19,58 ± 5,91	0.016
LASct (%)	15,64 ± 5,32	19,16 ± 4,98	0.002

Values are presented as mean ± standard deviation. Abbreviations: *T2DM,* type 2 diabetes mellitus; *LVMI,* left ventricular mass index; *LAVI,* left atrial volume index; *LASr,* left atrial reservoir function; *LAScd,* left atrial conduit function; *LASct,* left atrial contractile function

## Discussion

In our study, LA reservoir, conduit, and contraction functions, as measured by STE in healthy individuals, were found to be 38.75% ± 5.43%, 19.58% ± 5.91%, and 19.16% ± 4.98%, respectively. A meta-analysis conducted in 2017, comprising 40 studies and 2542 healthy subjects, reported the average LA reservoir, conduit, and contraction functions in healthy individuals as 39%, 23%, and 17%, respectively [18]. The NORRE study, conducted by the European Society of Cardiac Radiology in 2018, encompassing 371 subjects and utilizing STE, reported corresponding LA functions as 42.5%, 25.7%, and 16.3% [19]. Additionally, a multicenter study in Korea in 2020 revealed normal reservoir, conduit, and contractile (pump) functions in healthy individuals to be 35.9% ± 10.6%, 21.9% ± 9.3%, and 13.9% ± 3.6%, respectively [20]. In summary, the normal values of LA functions measured by STE exhibit variation across scientific publications, influenced by factors such as sample size, equipment, software, and age [21].

In our study, the values for the reservoir, conduit, and contractile functions in the group of patients with T2DM were recorded as 31.2% ± 4.56%, 14.77% ± 6.3%, and 16.36% ± 4.82%, respectively. These results exhibited a notable decrease compared to the control group, and these differences were statistically significant, denoted by *p* < 0.05. Consistent findings from both clinical and preclinical studies consistently suggest that T2DM is independently associated with cardiac fibrosis. The widespread fibrosis and stiffening of cardiac muscle can lead to disruptions in LA function and LV diastolic function. The mechanisms underlying diabetes-related fibrosis may involve a combination of factors, including oxidative stress, inflammation, increased production of advanced glycation end-products (AGEs), and upregulation of growth factors [22].

In mice with T2DM, oxidative stress and inflammation resulting from prolonged exposure to hyperglycemia have been shown to enhance the expression of transforming growth factor-beta (TGF-β), activating functional signaling pathways. Moreover, the heightened production of Advanced Glycation End-products (AGEs) and AGE receptors contributes to overall cardiac fibrosis, as growth factor-regulated connective tissue undergoes enhanced modulation. Additionally, the activation of the renin-angiotensin-aldosterone system is implicated in promoting fibrosis through the TGF-β signaling pathway. The fibrosis observed in the LA of T2DM patients impairs LA functions [22]. T2DM may also be associated with alterations in electrophysiology. Some animal studies have indicated that T2DM increases conduction time between the two atria, enhances effective refractory period dispersion in the atria, and prolongs action potential duration [22, 23].

In our study, LA strain in T2DM patients, even in the absence of LV morphological abnormalities based on LVMI, was significantly reduced compared to the control group. A study by Jeremy M. Steele et al. (2020) involving 331 adolescents and young adults with a median age of 22.1 years, demonstrated a decrease in LA function using STE among patients with T2DM in the absence of LV structural changes [24]. Importantly, alterations in LA function assessed by STE were found to precede changes in LV structure measured by conventional LVMI.

Recent studies prefer assessing atrial functional changes using STE to early evaluate the effectiveness of treatment interventions. Data from the DIASTOLIC trial investigating the impact of lifestyle intervention on LA function in T2DM patients show the effectiveness of the intervention on LA function before any LV structural changes [25]. Kirsten Thiele et al.’s 2023 study revealed that empagliflozin improves LA function even before LV structural changes occur [26].

In our study, LA strain in T2DM patients, even in the absence of abnormal LA morphology based on LAVI, displayed a significant reduction compared to the control group. Sergio Mondillo et al. in 2011 reported a decrease in LA functional indices measured by STE in subjects with normal LAVI [27]. In the investigation conducted by Alain Patrick Menanga et al. (2020) on the structure and function of LA in T2DM patients, although the average value of LAVI (22 ± 6 mL/m^2^) fell within the normal reference range, the LA function indices measured by STE were notably reduced, particularly in terms of conduit and contractile functions [28].

An elevated LAVI is commonly regarded as a surrogate marker for increased pressure overload and chronicity, frequently utilized in clinical practice to assess diastolic dysfunction. In Tatjana Miljkovic et al.’s 2022 study, LAVI was identified as a significant predictor of diastolic dysfunction with increased LA pressure with an area under the curve (AUC) of 0.689 and a set cutoff value of 39.90 with a sensitivity of 75.0% and specificity of 66.3% [29]. Nevertheless, recent studies suggest that functional impairment may manifest even in individuals with a normal size of the LA, and LAVI exhibits low sensitivity in the early detection of LA dysfunction. Morris et al. study (2018) involving 517 patients at risk of diastolic dysfunction, LA strain showed higher sensitivity than LAVI in detecting patients with diastolic dysfunction, with a cutoff point for LA strain < 23% yielding a sensitivity of 73% and specificity of 76% for identifying diastolic dysfunction when LAVI was normal. Morris et al. recognized that incorporating LA strain into the LAVI algorithm could enhance the ability to detect early-stage diastolic dysfunction [30].

Building on our study and the work of global colleagues, it is anticipated that future diagnostic and treatment guidelines from professional societies will incorporate LA functions assessed by STE. Particularly, STE technology remains uncommon in Central Vietnam. Based on the findings of our study, we conclude that there is a need for investment in implementing STE into clinical practice in Central Vietnam. This can contribute to the early detection of LA function changes in patients with T2DM.

## Limitations

This single-center study is constrained by a small sample size, highlighting the necessity for multicenter research with a larger sample size to garner more robust evidence for clinical applications. Furthermore, as the study adopts a cross-sectional design, it can only establish correlations rather than infer causal relationships. Longitudinal studies are required to address this issue. The research is conducted only on one type of ultrasound machine and software. A comparison among software from different manufacturers has not been performed.

## Conclusions

STE of the LA demonstrates a significant reduction in reservoir, conduit, and contractile functions in the T2DM group compared to the control group (*p* < 0.05). These functions are also diminished in T2DM patients without increased LAVI and LVMI.

## Data Availability

The datasets used and/or analyzed during the current study are available from the corresponding author on reasonable request.

## Abbreviations

T2DM: Type 2 diabetes mellitus
LA: Left atrial
STE: Speckle tracking echocardiography
LASr: Left atrial reservoir strain
LAScd: Left atrial conduit strain
LASct: Left atrial contractile strain
LAVI: Left atrial volume index
LVMI: Left ventricular mass index
LV: Left ventricle
FPG: Fasting plasma glucose
OGTT: Oral glucose tolerance test
HbA1c: Hemoglobin A1c
BSA: Body surface area
BMI: Body mass index
WC: Waist circumference
SBP: Systolic blood pressure
DBP: Diastolic blood pressure
LVM: Left ventricular mass
LVIDd: Left ventricular internal diameter in diastole
IVSd: Interventricular septum thickness in diastole
LVPWd: Left ventricular posterior wall thickness in diastole
LAd: Left atrial diameter
HDL-C: High-density lipoprotein cholesterol
LVEF: Left ventricular ejection fraction
TGF-β: Transforming growth factor-beta
AGEs: Advanced glycation end-products
